# Optimizing myopia prediction in children and adolescents using machine learning: a multi-factorial risk assessment model

**DOI:** 10.3389/fmed.2025.1672432

**Published:** 2025-11-13

**Authors:** Yue Xi, Wei Zhu, Wenjing Yan, Xiaobin Wei, Qian Zhao, Shengting Dai

**Affiliations:** 1 School of Physical Education, Shanghai University, Shanghai, China; 2 Yinchuan Maternal and Child Health Hospital, Yinchuan, China; 3 School of Physical Education, Shanxi Normal University, Taiyuan, Shanxi, China; 4 Division of Sports Science and Physical Education, Tsinghua University, Beijing, China; 5 The No. 10 Primary School of Zhongning County, Zhongwei, China; 6 School of Sports Science and Engineering, East China University of Science and Technology, Shanghai, China

**Keywords:** children and adolescents, myopia, modifiable risk factors, LightGBM, machine learning

## Abstract

**Background:**

Most previous studies on myopia in children and adolescents have primarily focused on genetic and environmental factors. This study aimed to explore modifiable behavioral, sociodemographic, and psychological contributors to myopia and to evaluate the potential of machine learning (ML) models in identifying at-risk individuals.

**Methods:**

A cross-sectional survey was conducted in eight primary and secondary schools in a Chinese province between October and December 2023. The dataset was split into training and testing sets (7:3). LASSO regression identified potential predictors, followed by multivariate logistic regression to determine independent risk factors. Ten machine learning algorithms were used to build prediction models: logistic regression, support vector machine (SVM), gradient boosting machine (GBM), neural network (NNET), extreme gradient boosting (XGBoost), k-nearest neighbors (KNN), random forest adaptive boosting (AdaBoost), LightGBM, and CatBoost. Model performance was evaluated using accuracy, F1 score, specificity, sensitivity, and area under the receiver operating characteristic (ROC) curve (AUC). SHapley Additive exPlanations (SHAP) were used to interpret variable contributions in the best-performing model.

**Results:**

The study included 2,086 children and adolescents (mean age 9.8 ± 2.7 years; 50.5% female), with an overall myopia prevalence of 25.12%. Independent risk factors for myopia included parental myopia, only-child status, physical activity level, mother’s education level, age, and physical activity behavior. Among all models, the LightGBM algorithm achieved the best predictive performance (AUC = 0.738, 95% CI: 0.709–0.767). SHAP analysis identified parental myopia, physical activity level, only-child status, and physical activity behavior as the most influential predictors.

**Conclusion:**

Although ML models showed limited predictive accuracy, they helped identify modifiable risk factors associated with childhood and adolescent myopia. These findings may inform the design of targeted prevention strategies and early behavioral interventions rather than serve as clinical diagnostic tools.

## Introduction

Myopia is an increasingly severe public health issue worldwide and is particularly prominent among children and adolescents (1, 2). In recent years, with changes in the social environment and lifestyle, the incidence of myopia has significantly increased, especially in Asian countries, where the prevalence of myopia among children and adolescents has reached alarming levels (3). Myopia not only affects the quality of life of children but also may lead to a range of eye health issues, such as fundus lesions and retinal detachment, and even increases the risk of blindness in adulthood (4).

Importantly, extensive research has confirmed that prolonged lack of outdoor activities and physical exercise not only impacts physical health but also creates conditions conducive to the development of myopia. The dopamine hypothesis explains that outdoor light stimulates retinal dopamine release, inhibiting axial elongation (5, 6). Studies like Rai et al. have demonstrated clear urban–rural and gender gradients in myopia prevalence, confirming light exposure rather than demographics as the key protective factor (7). Together with near work and peripheral defocus theories (8, 9), these mechanisms highlight the multifactorial nature of myopia development. The overindulgence and high academic expectations often present in only-child families may increase children’s eye strain and psychological stress, further promoting the onset of myopia (10). Thus, beyond genetic predisposition, environmental and behavioral factors—including family dynamics—play crucial roles in myopia development.

Beyond these traditional factors (11), an increasing number of studies have focused on the relationship between myopia and mental health. Psychological stress (12, 13), anxiety/depression (14, 15), and other factors are considered important psychological factors affecting eye health. Excessive academic and environmental pressure may exacerbate the progression of myopia. Moreover, the occurrence of myopia is often accompanied by abnormal body posture (16), changes in personality traits (17), and emotional changes (18), further illustrating the significant impact of mental health on myopia. Therefore, in addition to traditional environmental and behavioral factors, understanding the role of psychological factors will help researchers comprehensively grasp the mechanisms of myopia development and facilitate more effective intervention strategies.

In recent years, machine learning has been widely applied in myopia research, particularly in the prediction of axial length, myopia risk assessment, and the identification of factors influencing myopia (19, 20). Machine learning can handle complex, multidimensional datasets and reveal nonlinear relationships that traditional statistical methods struggle to capture, providing new perspectives for accurate prediction and early screening of myopia (20, 21). Therefore, this study aims to apply machine learning approaches to identify key modifiable risk factors associated with myopia among children and adolescents, focusing on a wide range of predictors including demographic characteristics, genetic background, physical activity, psychological stress, screen-related behavior, dietary patterns, sleep routines, and academic pressure. Rather than solely optimizing prediction accuracy, we seek to quantify the relative contribution of these variables to myopia risk, particularly those behavioral and psychological factors that can be improved through interventions, thereby providing evidence-based guidance for public health prevention strategies.

## Methods

### Subject of investigation

This study was conducted from October to December 2023 in eight primary and secondary schools located in a prefecture-level city in a certain province of China, and involved vision screening and accompanying questionnaire surveys. In accordance with the inclusion criteria (including cooperation with the survey, no eye diseases during the survey period, and no history of keratoconus treatment), data were collected from 2,112 participants. The study included 17 features, such as student demographics, parental information, physical activity level, lifestyle behaviors, generalized anxiety, and academic pressure. Prior to the study, the purpose and procedures of the research were thoroughly explained to the parents or legal guardians, and a written informed consent form, approved by the Ethics Committee of East China Normal University and adhering to the Declaration of Helsinki, was signed before the research commenced (HR476–2020).

### Examination items

#### Questionnaire survey

The questionnaire included personal information about the students (such as gender, age, grade, place of residence, and myopia status), family information (such as number of children, myopia status, education level, parental occupation, and household income), lifestyle factors (dietary habits, sleep behaviors, exercise behaviors), the International Physical Activity Questionnaire (IPAQ, short version), the Generalized Anxiety Scale, and the Sources of Academic Stress for Middle School Students Questionnaire.

#### The international physical activity questionnaire (IPAQ, short version)

The IPAQ was used to assess physical activity levels. According to the Guidelines for Data Processing and Analysis of the International Physical Activity Questionnaire by the World Health Organization and the calculation principles proposed by Fan Mengyu (22), physical activity levels are categorized into three groups—low, moderate, and high—on the basis of the frequency and duration of various types of physical activity within a week. MET-minutes were calculated using the formula: MET intensity × duration (minutes) × frequency (days/week). High level: Participants engage in vigorous-intensity physical activity at least 3 days per week and accumulate a total of at least 1,500 MET-minutes per week, or they engage in transportation, moderate, and/or vigorous-intensity physical activities for 7 or more days, accumulating at least 3,000 MET-minutes per week. Moderate level: Participants engage in vigorous-intensity physical activity for at least 3 days, with at least 20 min of activity per day, or moderate-intensity physical activity for at least 5 days, with at least 30 min of activity per day, or they engage in transportation, moderate-, and/or vigorous-intensity physical activity for 5 or more days, accumulating at least 600 MET-minutes per week. Low level: Participants reported no physical activity, or the level of physical activity did not meet the criteria for moderate and high levels.

#### Generalized anxiety disorder 7 (GAD-7)

The GAD-7 is a brief self-assessment scale (23) consisting of 7 items. Each item is rated on a 4-point scale: 3 = nearly every day; 2 = more than half the days; 1 = several days; 0 = not at all. The total score is the sum of the scores for all 7 items, with a range of 0–21 points. The following cutoff points were used to categorize anxiety levels: 0–4 (minimal/no anxiety), 5–9 (mild anxiety), 10–14 (moderate anxiety), and 15–21 (severe anxiety).

#### Sources of academic stress for middle school students (SSS)

This scale was developed by Chen Xu (24) in 2004 and uses a five-point Likert scale, with ratings ranging from 1 to 5 corresponding to “no stress,” “slightly stressful,” “moderately stressful,” “highly stressful,” and “extremely stressful,” respectively. A higher score indicates greater stress.

#### Lifestyle questionnaire

Lifestyle behavior information was collected via a frequency survey designed by the research team. This questionnaire contains simple questions to gather data on the lifestyle behaviors of children and adolescents. It covers four behavior areas: eye-related behavior, sleep behavior, dietary habits, and physical activity behavior. Examples include “In the past month, how much time did you spend watching TV after school on school days?” with 5 options: “None, Half an hour, One hour, Less than two hours, More than two hours”; “In the past month, how many hours of sleep (including naps) did you get daily?” with 2 options: “8 h or more, Less than 8 h”; “In the past month, how often did you pay attention to balanced nutrition in your diet?” with 4 options: “Never, Occasionally, Frequently, Always”; “In the past month, how did you feel after physical education classes?” with 3 options: “Sweaty and tired, Sweaty but relaxed, Not sweaty and relaxed.”

#### Myopia

Myopia status was determined through a parental questionnaire in which parents or legal guardians were asked to report whether their child had been diagnosed with myopia by a medical professional. The specific item was: “Has your child been diagnosed with myopia by an eye doctor?”

### Data processing

In this study, a questionnaire was used to collect student information. Prior to completing the questionnaire, the researchers provided instructions on how to fill it out, and the class teachers supervised the completion and collection of the questionnaires to ensure their quality. A total of 2,112 participants’ data were collected. During the data preprocessing process, blank samples were removed, and missing values in some samples were filled in. Ultimately, 2,086 samples were retained. The dataset was then divided into a training set (*n* = 1,461) and a test set (*n* = 625) at a 7:3 ratio.

### Myopia prediction model

Variable selection and model construction were performed using the training set data. First, univariate logistic regression was applied to the training set to identify key factors related to myopia. Various machine learning (ML) algorithms, including the support vector machine (SVM), gradient boosting machine (GBM), neural network (NNET), extreme gradient boost (XGBoost), K-nearest neighbor (KNN), random forest adaptive boosting (AdaBoost), light gradient boosting machine (LightGBM), and categorical boosting (CatBoost) algorithms, were subsequently used to build prediction models.

In the model construction process, each machine learning algorithm was first used to build classification models on the basis of the training set, with automatic parameter optimization used to determine the best hyperparameters for each model. Next, the models were evaluated on the test set, and the best model was selected on the basis of various performance metrics. The area under the curve (AUC) was used as the primary evaluation metric to automatically compare the predictive performance of different models, and receiver operating characteristic (ROC) curves were generated to display each model’s performance on the test dataset. Additionally, other evaluation metrics, were used including accuracy, F1 score, specificity, and sensitivity. These metrics provide a comprehensive reflection of the model’s performance across different aspects and help to assess the effectiveness and stability of the model.

Furthermore, we used R to calculate the Shapley values of each influencing factor in the LightGBM model, measuring the contribution of each feature to the prediction results. By calculating the mean absolute Shapley value for each variable across all the measurements and ranking them, we further assessed the feature importance in the LightGBM model. The greater the feature importance is, the greater its impact on the occurrence of myopia. In the Shapley value visualization, each point represents a sample, and the color intensity reflects the importance of the feature, with yellow indicating high importance and purple indicating low importance.

## Results

### General situation

A total of 2,086 children and adolescents aged 6 to 16 years (9.8 ± 2.7 years) were included in this study. Among them, 1,032 were male (49.5%), and 1,054 were female (50.5%). There were 1,697 primary school students (81.3%) and 389 middle school students (18.7%). A total of 524 students were diagnosed with myopia, yielding a myopia rate of 25.12%. Among these, 290 boys had myopia (55.4%), and 234 girls had myopia (44.6%), with a statistically significant difference between genders (*χ*^2^ = 9.95, *p* = 0.002). In terms of grade level, 345 primary school students had myopia (20.33%), and 179 middle school students had myopia (46.01%).

The distribution of physical activity levels was as follows: 354 students (17.0%) were classified as high, 311 students (14.9%) were classified as moderate, and 1,421 students (68.1%) were classified as low. Overall, the physical activity levels of the children and adolescents in the study were relatively low. The anxiety score was 2.71 ± 4.32. In terms of anxiety severity, 1,579 students (75.6%) had normal levels of anxiety, 351 students (16.8%) had mild anxiety, 75 students (3.6%) had moderate anxiety, 50 students (2.4%) had moderate-to-severe anxiety, and 31 students (1.5%) had severe anxiety. The academic stress score was 149.40 ± 57.83. In terms of stress severity, 234 students (11.2%) reported no stress, 518 students (24.8%) reported mild stress, 450 students (21.6%) reported moderate stress, 848 students (40.7%) reported moderate-to-severe stress, and 36 students (1.7%) reported extreme stress.

The study results show that anxiety and academic stress levels are relatively high among children and adolescents, which should be a cause for concern for relevant authorities. The detailed characteristics are shown in Table 1, and no statistically significant differences were found between the training and testing sets.

**Table 1:** Baseline characteristics of the study cohort.

Variables	Training set (*N* = 1,461)	Testing set (*N* = 625)	*p* value
Personal information			
Myopia			0.999
Yes	367	157	
No	1,094	468	
Age	9 (6–16)	9 (6–16)	0.827
Gender			0.908
Male	724	308	
Female	737	317	
Residence			0.566
Countryside	372	172	
Townships	159	73	
County town	867	350	
Municipal	63	30	
Stage			0.504
Primary school	1,194	503	
Middle school	267	122	
Family information			
Number of children			0.758
Only child	249	110	
Multiple children	1,212	515	
Family income (monthly/RMB)			0.042
Under 4,000	493	238	
4,000–5,999	467	203	
6,000–7,999	270	83	
8,000–9,999	126	51	
10,000 and above	105	50	
Parental occupation			0.265
Self-employed	295	109	
Intellectuals/cadres	72	34	
Worker	342	131	
Farmers	493	236	
Other	259	115	
Parental myopia			0.954
Yes	503	216	
No	958	409	
Father’s level of education			0.329
Below high school	1,057	436	
University and above	210	91	
Unknown	194	98	
Mother’s level of education			0.165
Below high school	1,011	420	
University and above	242	96	
Unknown	208	109	
Lifestyle behavioral factors			
Physical Activity Level			0.091
High level	247	107	
Medium level	202	109	
Low level	1,012	409	
Eating behavior	9.20 (2.10)	9.14 (2.07)	0.445
Sleep behavior	7.89 (1.38)	7.93 (1.35)	0.423
Exercise behavior	4.48 (0.76)	4.44 (0.81)	0.054
Eye-related Behavior	24.81 (2.66)	24.69 (2.83)	0.521
Psychological factor			
Anxiety level	2.61 (4.19)	2.92 (4.62)	0.016
Level of academic stress	148.93 (58.34)	150.49 (56.65)	0.317

### Model performance and comparisons

A total of 17 variables were collected on the basis of the inclusion criteria. Through LASSO regression analysis, six variables associated with myopia were selected: physical activity level, mother’s education level, parental myopia status, gender, only child status, and physical activity behavior. These predictive variables were then included in both univariate and multivariate logistic regression analyses. The results indicated that children with myopic parents had a significantly greater risk of myopia (OR = 3.13, 95% CI: 2.27–4.17). Similarly, only child status was associated with a greater risk of myopia (OR = 1.71, 95% CI: 1.15–2.55), and increasing age was also linked to a greater likelihood of myopia (OR = 1.78, 95% CI: 1.60–1.99). Low physical activity levels and insufficient physical activity were significantly associated with an increased risk of myopia (OR = 0.67, 95% CI: 0.47–0.96; OR = 0.71, 95% CI: 0.60–0.85). Additionally, children with a higher maternal education level had a significantly increased risk of myopia (OR = 1.56, *p* = 0.039). The details are presented in Table 2.

**Table 2:** Multivariate logistic regression analysis for identifying independent risk factors.

Variables	Univariable		Multivariable	
	OR (95% CI)	*p* value	OR (95% CI)	*p* value
Gender				
Male				
Female	0.75 (0.59–0.95)	0.015	0.76 (0.58–1.00)	0.052
Stage				
Primary school				
Middle school	3.46 (2.62–4.58)	<0.001	0.66 (0.40–1.10)	0.110
Residence				
Countryside				
Townships	1.84 (1.24–2.75)	0.003	1.01 (0.64–1.61)	0.962
County town	0.88 (0.66–1.17)	0.392	1.23 (0.81–1.86)	0.336
Municipal	1.42 (0.79–2.53)	0.241	1.47 (0.75–2.89)	0.268
Number of children				
Only child				
Multiple children	1.54 (1.10–2.17)	0.013	1.71 (1.15–2.55)	0.008
Family income (monthly/RMB)				
Under 4,000				
4,000–5,999	1.14 (0.85–1.51)	0.386		
6,000–7,999	0.93 (0.66–1.32)	0.702		
8,000–9,999	0.90 (0.57–1.43)	0.653		
10,000 and above	0.84 (0.51–1.40)	0.510		
Parental myopia				
Yes				
No	2.08 (1.64–2.70)	<0.001	3.13 (2.27–4.17)	<0.001
Parental occupation				
Self-employed				
Intellectuals/cadres	1.52 (0.85–2.71)	0.159	0.86 (0.42–1.78)	0.687
Worker	1.52 (1.06–2.19)	0.023	0.97 (0.63–1.48)	0.879
Farmers	1.08 (0.76–1.53)	0.656	0.83 (0.54–1.27)	0.388
Other	1.39 (0.94–2.05)	0.097	1.21 (0.77–1.90)	0.401
Physical activity level				
High level				
Medium level	1.12 (0.75–1.67)	0.575	0.89 (0.57–1.40)	0.616
Low level	0.62 (0.46–0.84)	0.002	0.67 (0.47–0.96)	0.027
Father’s level of education				
Below high school				
University and above	0.89 (0.63–1.26)	0.504		
Unknown	0.79 (0.54–1.14)	0.199		
Mother’s level of education				
Below high school				
University and above	1.40 (1.03–1.91)	0.033	1.56 (1.02–2.38)	0.039
Unknown	1.03 (0.73–1.46)	0.858	0.87 (0.57–1.32)	0.500
Age	1.51 (1.42–1.62)	<0.001	1.78 (1.60–1.99)	<0.001
Anxiety level	1.01 (0.99–1.04)	0.333		
Sleep behavior	0.95 (0.88–1.04)	0.284		
Eating behavior	0.97 (0.92–1.02)	0.253		
Exercise behavior	0.70 (0.60–0.81)	<0.001	0.71 (0.60–0.85)	*p* < 0.001
Eye-related Behavior	0.97 (0.93–1.01)	0.105		
Level of academic stress	1.00 (1.00–1.00)	0.084		

**Figure 1:**
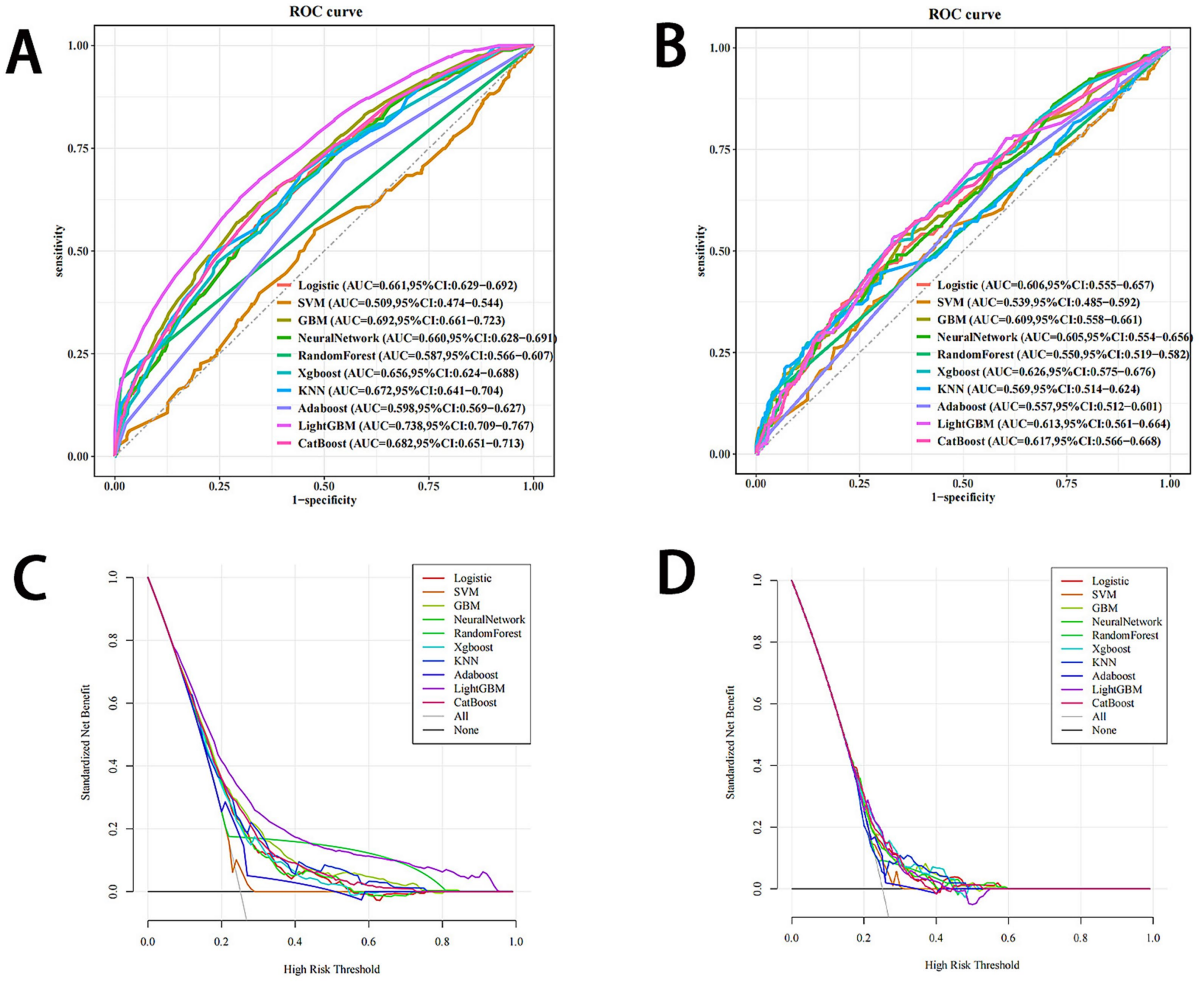
Comprehensive evaluation of machine learning models. **(A)** ROC curves and AUC values of the training set. **(B)** ROC curves and AUC values of the testing set. **(C)** Decision curve analysis of the logistic, SVM, GBM, neural network, Xogboost, KNN, Random forest AdaBoost, LightGBM, and CatBoost models in the training set. **(D)** Decision curve analysis of the logistic, SVM, GBM, neural network, Xogboost, KNN, Random forest AdaBoost, LightGBM, and CatBoost models in the testing set. ROC, receiver operating characteristic; AUC, area under the receiver operating characteristic curve; logistic, logistic regression; VM, support vector machine; GBM, gradient boosting machine; Xgboost, extreme gradient boosting; KNN, k-nearest neighbors; AdaBoost, adaptive boosting; LightGBM, light gradient boosting machine; CatBoost, categorical boosting.

On the basis of the selected independent risk factors—physical activity level, mother’s education level, parental myopia status, gender, only child status, and physical activity behavior—ten machine learning models were constructed to predict the risk of myopia in children and adolescents. These models included logistic regression, SVM, GBM, NNET, XGBoost, KNN, Random forest, AdaBoost, LightGBM, and CatBoost. The performance comparison of each model is shown in Figure 1. The results indicate that the LightGBM model had the highest AUC value in the training set (AUC = 0.738, 95% CI: 0.709–0.767).

In addition, the five performance metrics for the LightGBM model in the training set were as follows: accuracy = 0.683, sensitivity = 0.629, specificity = 0.701, F1 score = 0.499, as shown in Table 3. In the testing set, the five performance metrics for LightGBM were as follows: accuracy = 0.634, sensitivity = 0.535, specificity = 0.667, F1 score = 0.423. Overall, the LightGBM model exhibited the best performance.

**Table 3:** Comparison of performance metrics across models.

Model	Accuracy	Sensitivity	Specificity	F1 score
Training set				
Logistic	0.575	0.689	0.537	0.449
SVM	0.53	0.55	0.523	0.37
GBM	0.674	0.569	0.708	0.467
NeuralNetwork	0.631	0.583	0.647	0.443
Xgboost	0.684	0.471	0.756	0.429
KNN	0.697	0.496	0.764	0.451
RandomForest	0.668	0.545	0.705	0.445
AdaBoost	0.52	0.719	0.452	0.429
LightGBM	0.683	0.629	0.701	0.499
CatBoost	0.631	0.64	0.628	0.466
Testing set				
Logistic	0.686	0.369	0.793	0.372
SVM	0.637	0.363	0.729	0.334
GBM	0.621	0.535	0.65	0.415
NeuralNetwork	0.629	0.471	0.682	0.389
Xgboost	0.632	0.522	0.669	0.416
KNN	0.712	0.299	0.85	0.343
RandomForest	0.635	0.515	0.675	0.415
AdaBoost	0.485	0.688	0.417	0.401
LightGBM	0.634	0.535	0.667	0.423
CatBoost	0.605	0.573	0.615	0.422

### Model interpretations

**Figure 2:**
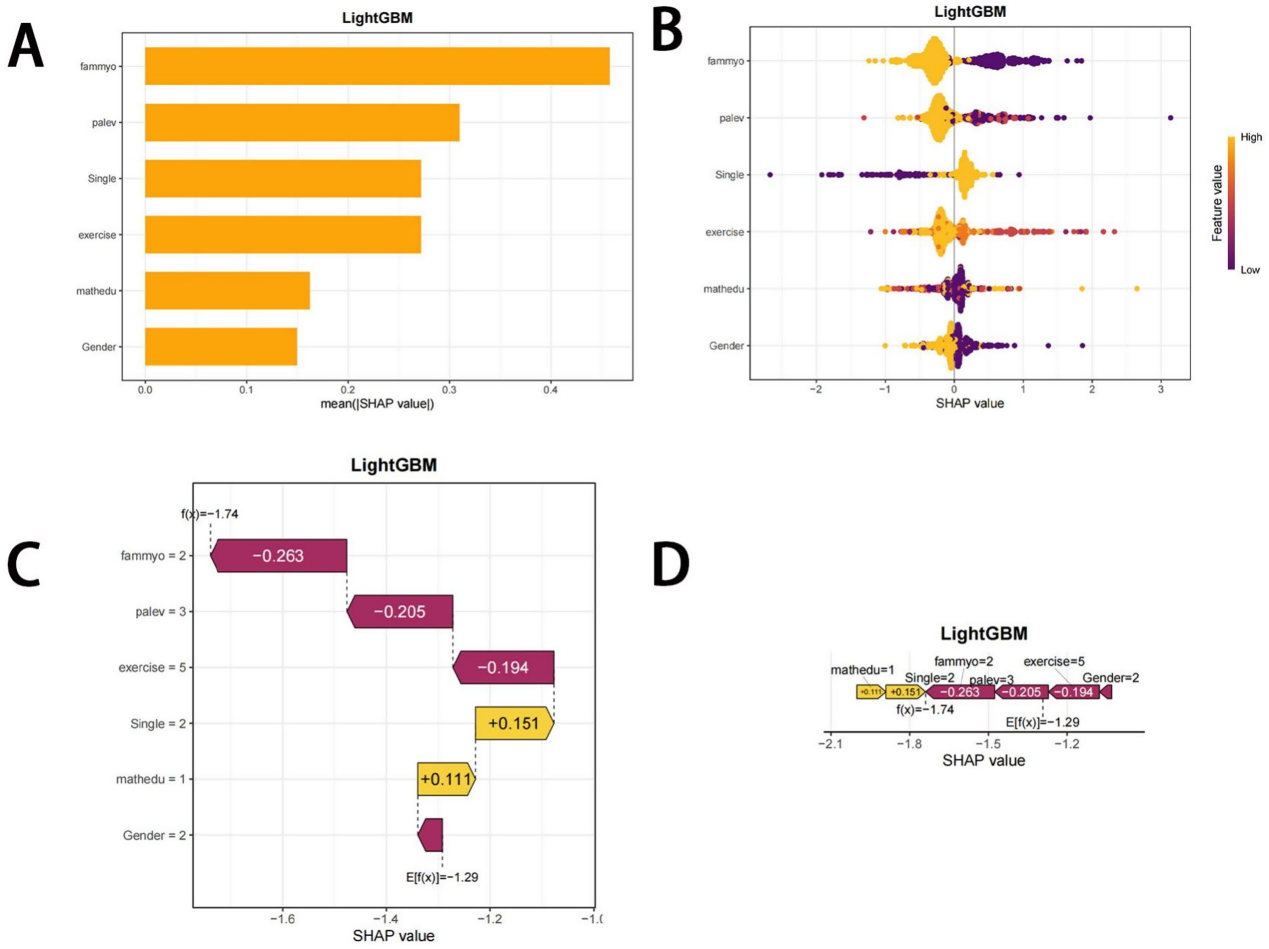
SHAP plots. **(A)** Bar chart of the mean absolute SHAP value for each predictor of the LightGBM model in descending order. **(B)** SHAP summary plot showing feature importance for each predictor of the LightGBM model in descending order. The upper predictors are more important to the model’s predictive outcome. A dot is created for each feature attribution value for the LightGBM model of each student. The further away a dot is from the baseline SHAP value of zero, the stronger its effect on the model output. The dots are colored according to the values of the features. Yellow represents higher feature values, and red represents lower feature values. **(C)** Consent waterfall plot showing an example of interpretability analysis for a student. The yellow part of the feature value represents a positive effect on the model. The deep red part of the feature value represents a negative effect on the model **(D)**. The force plots provide personalized feature attributions via two representative examples. SHAP, Shapley additive explanations.

The contributions of the predictive factors to the prediction results were quantified via SHAP (SHapley Additive exPlanations). SHAP applies a game-theory-based method to evaluate the importance of each feature. SHAP significance analysis via the LightGBM model visualized the ranking of feature importance, as shown in Figure 2A. Our analysis identified the top six risk factors associated with myopia: parental myopia status, physical activity level, only child status, physical activity behavior score, mother’s education level, and gender.

The SHAP summary plot (Figure 2B) further supplements this ranking by visually displaying the impact of each feature on the model’s output. A positive Shapley value for each feature indicates increased risk, whereas a negative value suggests decreased risk. To further illustrate the application of the LightGBM model, we randomly selected an individual from the validation cohort. The waterfall plot displays the feature contributions for this individual, with yellow and purple bars representing the contributing features to myopia risk prediction (Figures 2C,D).

These visualizations help relevant stakeholders quickly understand which factors are most strongly associated with the increase or decrease in myopia risk among children and adolescents. The results show that parental myopia, low physical activity level, and only child status are the top three factors contributing to the prediction of myopia risk, with Figures 2C,D demonstrating the specific impact of these factors on individual myopia predictions.

## Discussion

This study explored predictors of myopia among children and adolescents in a province of China using various machine learning algorithms. Key predictors included parental myopia, gender, maternal education level, only-child status, general physical activity levels, and specific school-based physical activity behaviors. These findings are consistent with prior research and underscore the multifactorial nature of myopia development, involving both genetic predispositions and modifiable lifestyle patterns.

This study included 2,086 children and adolescents aged 6 to 16 years. The results revealed that the overall myopia rate among students was 25.12%, of which 55.4% were boys and 44.6% were girls. The results revealed that the overall myopia rate among students was 25.12% with notable differences between middle school students (85.24%) and primary school students (25.52%). This finding further confirms the high incidence of myopia among children and adolescents in recent years, particularly among middle school students (25, 26). Additionally, physical activity level, anxiety level, and academic pressure have been identified as important factors influencing myopia risk (15, 27, 28). Our data show that children and adolescents generally have low levels of physical activity, along with high levels of anxiety and academic pressure, which is consistent with studies both domestically and internationally (29). These results suggest that the health behaviors, psychological state, and academic pressure of children and adolescents are closely related to the onset of myopia, highlighting the importance of early and comprehensive intervention strategies.

Through LASSO regression, we identified parental myopia status, only child status, physical activity level, maternal education level, age, and physical activity behavior as independent risk factors for myopia in children and adolescents, which is consistent with several studies. Specifically, genetic factors have been shown to significantly influence myopia risk, with adolescents who have a family history of myopia being at greater risk than those with parents who do not have myopia (30). Additionally, age is positively correlated with the occurrence of myopia, and the myopia rate increases significantly with age (31). Notably, this study confirms that low physical activity levels and insufficient physical activity behavior are significantly associated with a greater risk of myopia (32), with physical activity behavior primarily referring to activities during physical education classes, which further emphasizes the important role of school-based physical education in myopia prevention. On the other hand, this finding also supports the concept proposed by Yan Jinhui (33) regarding the combined effect of “outdoor activities” and “exercise” in myopia prevention. Moreover, children from only child families and those with higher maternal education levels face a greater risk of myopia. Research by Quan Xiaojun (10) revealed that only children have a higher myopia rate than nononly children do, and the risk of myopia increases with higher maternal education. This may be due to the stronger influence of the mother’s education level on the child’s education level than the father’s education level does (34). Compared with nononly children, only children tend to receive more attention from their parents, particularly those with a greater likelihood of attending extracurricular classes, which in turn increases the risk of myopia (35).

To more accurately predict myopia risk in children and adolescents and select the best predictive model, this study used the six feature variables selected by LASSO regression to build and compare nine machine learning models. By utilizing metrics such as accuracy, precision, F1 score, specificity, sensitivity, and area under the receiver operating characteristic curve (AUC), we provide a scientific and comprehensive evaluation framework for myopia risk prediction. The results revealed that the LightGBM model performed the best (training set: AUC 0.738; testing set: AUC 0.613). LightGBM is a gradient boosting decision tree-based machine learning algorithm designed for efficient model construction and training, suitable for large-scale and high-dimensional datasets, and widely applied in multiple fields, especially in medical diagnostics (36, 37). However, the importance ranking in the LightGBM model reflects only the overall influence of variables and does not express the role of variables in specific categories. Therefore, this study introduces the SHAP method to explain the importance and contribution of variables in the LightGBM model.

This study utilized SHAP (Shapley additive explanations) analysis to further uncover the contributions of key features, such as physical activity level, physical activity behavior score, parental myopia status, gender, and mother’s education level, in predicting myopia outcomes, thereby providing interpretable insights for myopia prevention strategies. These findings are highly consistent with the conclusions of the literature, further validating the importance of these factors in the onset of myopia. Our study revealed that parental myopia is crucial for predicting whether children and adolescents will develop myopia. A large body of research has confirmed that genetics is the most direct explanation for myopia, with children of myopic parents being at a greater risk of developing myopia (38). Moreover, genetic-environmental effects may also play a role, where parental behaviors and rearing practices increase myopia risk in children, with environmental factors acting as intermediaries in the genetic-myopia relationship (39). Notably, physical activity also plays a critical role in myopia. Our results show that physical activity level and physical activity behavior, particularly related to school sports activities, significantly contribute to predicting myopia risk in children and adolescents. These factors reflect students’ enthusiasm for participating in physical education classes and their physical exertion during these activities, demonstrating a close relationship between high levels of physical activity and lower myopia risk. These findings also highlight the strong role of outdoor activities and physical exercise in mitigating myopia. Extensive research has confirmed the significant effect of outdoor activities on reducing myopia risk (40, 41), primarily emphasizing the importance of daylight environments (42). Moreover, physical activities themselves, with their rich content and differentiated exercise methods, are considered to have a positive impact on promoting adolescent visual health and slowing myopia progression. Physical activity at different thresholds can have differential effects on myopia prevention. For example, long-duration (≥24 weeks), moderate-frequency (3–4 times/week), and short-duration (60–90 min) exercise regimens have been proven to be reference thresholds for positive effects (43). Physical activity promotes overall blood circulation, enhances muscle strength, regulates eye muscle function, and alleviates tension in the ciliary muscles, thus effectively slowing the progression of myopia (44). For example, sports such as table tennis can help relax the ciliary muscles and reduce the occurrence of accommodative myopia (pseudomyopia) (33). Furthermore, regular and intense physical activity can increase choroidal and ocular blood flow and stabilize ciliary body regulation, ensuring proper involvement of the choroid in refractive regulation, which guides the process of visual acuity development and promotes eye health (18, 45).

The contribution of physical activity and related behavioral scores to myopia risk was among the most prominent findings in this study. Children and adolescents with lower overall physical activity levels and less favorable activity behaviors were at increased risk of myopia, reinforcing the importance of modifiable lifestyle factors. SHAP analysis further highlighted the predictive contributions of only-child status, maternal education level, and gender. While maternal education emerged as a significant predictor across multiple models, the direction of its influence varied. This inconsistency may reflect a non-linear relationship shaped by interacting contextual factors. For instance, higher maternal education could imply increased health awareness and resources but may also correlate with heightened academic demands or increased screen exposure—both of which may negatively affect eye health. These findings point to the need for further research into the nuanced role of parental education in myopia development. Gender emerged as a significant predictor in our models, with females showing higher myopia risk, consistent with many international studies from East Asian populations (7). This gender difference may reflect multiple factors including behavioral patterns and outdoor activity levels. The consistent identification of this pattern across multiple machine learning algorithms validates its significance and demonstrates ML’s unique value: unlike traditional hypothesis-driven analyses constrained by international literature assumptions, our data-driven approach revealed population-specific patterns. This finding underscores that myopia interventions must be tailored to local behavioral contexts rather than following universal gender-based strategies.

These ML models could be integrated into clinical workflows as screening tools in pediatric clinics and schools. Healthcare providers could input basic information (parental myopia, physical activity levels) to generate instant risk assessments, enabling efficient triage and personalized prevention strategies. For clinicians, this provides evidence-based decision support and streamlines screening. For patients, it offers accessible risk assessment without specialized equipment. The SHAP visualizations help doctors explain specific risk factors to parents, improving communication and intervention compliance. Such tools are particularly valuable in resource-limited settings where access to eye care specialists is restricted.

The identification of unexpected patterns, such as the complex interplay between demographic and behavioral factors, exemplifies machine learning’s unique contribution to epidemiological research. Unlike hypothesis-driven approaches that may be constrained by existing assumptions, our machine learning models objectively identified risk patterns specific to our population. The convergence of multiple algorithms on similar predictors, despite their different underlying mechanisms, strengthens confidence in these findings. Furthermore, SHAP analysis provided transparent, interpretable insights into how each factor contributes to predictions, addressing common concerns about machine learning’s “black box” nature. Collectively, our results support the utility of machine learning models in identifying meaningful risk factors for myopia. SHAP-based interpretation underscores the critical role of physical activity, lending empirical support to the “environment-behavior interaction” hypothesis. From a public health perspective, these insights suggest that strengthening school-based physical activity programs may be a promising direction for myopia prevention strategies.

### Limitations and future directions

First, the study sample is primarily derived from a specific province in China, and the regional nature of the sample may limit the generalizability of the results, affecting their applicability to broader populations. Second, although the study collected a rich dataset through questionnaires, there may be biases in self-reported data, especially concerning sensitive issues such as behavior and mental health, which could affect the accuracy of the data. Additionally, this study did not include clinical indicators or physiological data, focusing primarily on modifiable behavioral factors, which may limit the comprehensive understanding of the mechanisms behind myopia. Finally, this study was designed as a single-center study and lacked external validation. Therefore, the reliability of the findings needs to be validated in other regions. Future research should explore the effects of different behavioral interventions, particularly personalized physical activity programs for children and adolescents, to improve the precision and effectiveness of myopia prevention. Longitudinal cohort studies will also help validate the causal relationships between these behavioral factors and myopia, advancing myopia prevention strategies in a more scientific and systematic direction.

## Conclusion

This study developed nine machine learning models based on six features selected via LASSO regression to explore risk factors associated with myopia in children and adolescents. Among these, the LightGBM model achieved the highest performance (AUC = 0.738 in training; 0.613 in validation), though overall predictive accuracy remained modest. These findings suggest that while machine learning offers potential for risk stratification and variable interpretation in myopia research, its current application for individualized prediction may be limited without further external validation.

## Data Availability

The original contributions presented in the study are included in the article/supplementary material, further inquiries can be directed to the corresponding author.
